# Understanding the anatomical basis of anorectal fistulas and their surgical management: exploring different types for enhanced precision and safety

**DOI:** 10.1007/s00595-025-02995-2

**Published:** 2025-01-31

**Authors:** Asim M. Almughamsi, Yasir Hassan Elhassan

**Affiliations:** 1 Department of Surgery, College of Medicine, Taibah University, Madinah, Saudi Arabia; 2 Department of Basic Medical Science, College of Medicine, Taibah University, Madinah, Saudi Arabia

**Keywords:** Anorectal fistula, Colorectal surgery, Anal anatomy, Sphincter preservation, Crohn's disease

## Abstract

Anorectal fistulas remain one of the most challenging conditions in colorectal surgery and require precise anatomical knowledge for successful management. This comprehensive review synthesizes the current evidence on the anatomical foundations of fistula development and treatment, particularly focusing on the cryptoglandular hypothesis and its clinical implications. A systematic analysis of the recent literature has examined the relationship between anatomical structures and fistula formation, classification systems, diagnostic modalities, and therapeutic approaches. The review revealed that anatomical considerations fundamentally influence treatment outcomes, with modern imaging techniques achieving up to 98% accuracy in delineating fistula anatomy. Key findings demonstrate that surgical success rates vary significantly based on anatomical complexity: 92–97% for simple fistulas versus 40–95% for complex cases using sphincter-sparing techniques. Emerging minimally invasive approaches and regenerative therapies, including mesenchymal stem cells, show promising results with 50–60% healing rates in complex cases. Special considerations are needed for complex cases such as Crohn's disease-related and rectovaginal fistulas. This review provides surgeons with an evidence-based framework for selecting optimal treatment strategies based on anatomical considerations, emphasizing the importance of preserving the anal sphincter function while achieving complete fistula eradication. Integrating advanced imaging, surgical techniques, and emerging therapies offers new possibilities for improving patient outcomes. This review aimed to bridge the gap between anatomical knowledge and practical surgical application, enhance clinical decision-making, and improve patient outcomes in anorectal fistula management.

## Introduction

For colorectal surgeons, anorectal fistulas represent one of the most technically demanding and intellectually challenging conditions to manage. Treatment success requires surgical skill and a deep understanding of anorectal anatomy and its variations. Anorectal fistulas are characterized by an abnormal epithelialized connection between the anal canal or rectum and the perianal skin [1]. Recent epidemiological data indicate an incidence of 8.6/100,000 people per year, which causes substantial morbidity, including chronic pain, recurrent infections, and an impaired quality of life. The cryptoglandular hypothesis is the most widely accepted theory for idiopathic cases, accounting for approximately 90% of anorectal fistulas [2]. For the practicing surgeon, three key challenges dominate clinical decision-making: accurate preoperative assessment of the fistula anatomy. Second, the appropriate surgical technique should be selected based on the anatomical configuration. Third, the sphincter function should be preserved while complete fistula eradication is achieved.

The management of anorectal fistulas has considerably evolved since ancient times. Contemporary surgical approaches have expanded beyond traditional fistulotomy to include sophisticated sphincter-sparing procedures, with technique selection heavily dependent on precise anatomical assessments. This evolution reflects a growing understanding of the critical balance between complete fistula eradication and preservation of anal continence. Recent studies have shown that inappropriate technique selection can lead to incontinence rates as high as 45% in complex cases. Parks' classification remains a cornerstone in surgical planning, categorizing fistulas into four types: intersphincteric, transsphincteric, suprasphincteric, and extrasphincteric [3]. This anatomical classification system directly guides surgical decision making, with each category presenting unique technical challenges and requiring specific surgical approaches. Understanding this classification is essential for selecting the most appropriate surgical technique and for minimizing postoperative complications.

Recent advancements in imaging techniques have significantly improved preoperative assessments. Modern imaging modalities, particularly high-resolution magnetic resonance imaging (MRI) and 3-dimensional (3D) endoanal ultrasound (EAUS), now provide unprecedented anatomical details, with recent studies demonstrating accuracies of up to 98% in delineating the fistula anatomy. This enhanced imaging capability allows surgeons to plan more precise and targeted interventions [4]. A recent meta-analysis showed that MRI has a sensitivity of 87% and specificity of 69% in identifying the primary fistula tract, whereas EAUS demonstrated a sensitivity of 87% and specificity of 43% [5]. For surgeons, these imaging capabilities directly translate into improved surgical planning and better patient outcomes, particularly in complex cases. Anorectal fistulas are particularly challenging in inflammatory bowel disease, especially Crohn's disease, where the cumulative incidence can reach 50% over 20 years [6]. The anatomical distortion and tissue friability associated with Crohn's disease require specific surgical considerations and often necessitate modification of the standard techniques. These cases often require a multidisciplinary approach that involves colorectal surgeons, gastroenterologists, and radiologists.

This review aimed to provide practicing surgeons with a detailed anatomical framework for understanding fistula pathogenesis, practical strategies for managing complex and recurrent cases, and updates on emerging technologies and their anatomical implications. Various surgical management techniques have been evaluated using an anatomical lens. Traditional methods, such as fistulotomy, have a success rate of 92–97% for simple fistulas but carry a risk of incontinence in up to 45% of cases of complex fistulas [7]. Emerging techniques and future directions in fistula management will be explored, including ligation of the intersphincteric fistula tract (LIFT) procedure, which has shown healing rates of 40–95% with minimal impact on continence [8, 9]. Bioprosthetic plugs and fibrin glue have gained attention as potential sphincter-sparing options, with success rates ranging from 24 to 92% [10]. One of the most exciting frontiers is the use of mesenchymal stem cells, with recent randomized controlled trials demonstrating complete healing in 50–60% of complex Crohn's perianal fistulas at 24 weeks [11, 12].

We emphasize the importance of a tailored approach to fistula management, based on individual patient anatomy and fistula characteristics. Extensive cohort studies have demonstrated improved outcomes with personalized treatment strategies [13]. Factors such as the location of the internal opening, complexity of the fistula tract, presence of secondary extensions, and the patient's underlying medical conditions play crucial roles in determining the most appropriate management strategy [14]. Postoperative care and follow-up are important aspects of management. Long-term follow-up is essential, as recurrence rates can be significant, ranging from 7 to 50%, depending on the type of fistula and surgical technique employed [15, 16].

By synthesizing the current evidence and highlighting the critical role of anatomy in successful fistula management, this review seeks to enhance clinical decision-making and ultimately improve patient outcomes in this challenging area of colorectal surgery. We aimed to provide clinicians with a comprehensive, evidence-based resource to bridge the gap between anatomical knowledge and practical surgical applications.

In conclusion, managing anorectal fistulas remains a complex and evolving field of colorectal surgery. A thorough understanding of the anatomical basis of fistula formation coupled with advances in imaging and surgical techniques has significantly improved our ability to treat these challenging conditions. However, an ideal management strategy still needs to be developed, particularly for complex fistulas and those associated with inflammatory bowel disease. As we refine the existing techniques and explore novel approaches, the importance of a personalized, anatomy-based approach to fistula management cannot be overstated. By focusing on the intricate relationship between fistula anatomy and surgical outcomes, we can continue to improve the care provided to patients with this challenging condition.

## Anatomy of the anorectum

### Foundation of fistula comprehension

Understanding anatomical variations and their surgical implications is crucial for successful management of anorectal fistulas. Although standard anatomical descriptions provide a framework, surgeons must appreciate the significant individual variations that can affect surgical approaches and outcomes. The intricate anatomy of the anorectum serves as a cornerstone for understanding the development, progression, and management of anorectal fistulas. Surgical success depends on recognition of both typical anatomy and common variations, particularly in the sphincter complex and anal gland distribution. This complex region, where form and function are inextricably linked, presents a unique challenge for both clinicians and surgeons. A profound grasp of this anatomy is not merely academic; it is the lens through which we view fistula pathogenesis, guide our diagnostic endeavors, and map our surgical interventions. The anorectum, the terminal portion of the gastrointestinal tract, is an evolutionary design that balances the conflicting demands of waste elimination and continence [17, 18]. Anatomical variations in this region can significantly affect the fistula development and surgical planning.

Several key variations have been identified in recent studies, as described below:

1. Sphincter complex variations
2. Internal anal sphincter thickness (normal range 1–3 mm, with significant individual variation)
3. External anal sphincter fiber arrangement (conventional triple-loop versus double-loop pattern)
4. Variable height of the intersphincteric space
5. Anal gland variations
6. Number (ranging from 4 to 10 per quadrant)
7. Depth of penetration into the sphincter complex
8. Distribution pattern around the circumference

The Anorectal Region comprises the anal canal and distal part of the rectum, each with its own specialized structures and functions. The anal canal, typically measuring 2–4 cm in length, extends from the anorectal junction to the anal verge. However, studies have shown that this length can vary by up to 50% among individuals, affecting the approach to fistula surgery and the risk of complications. This short yet complex passage is lined by an epithelium that undergoes a remarkable transition from columnar to squamous at the dentate line, a landmark of immense surgical significance. The dentate line's position can vary by ± 0.5 cm, affecting the location of anal glands and, consequently, the origin points of cryptoglandular fistulas. This variation must be considered during surgical planning, particularly in sphincter-sparing procedures.

The anal sphincter complex is located at the heart of the anorectal function, and its anatomical variations can significantly influence fistula development and surgical outcomes. Recent anatomical studies have identified three distinct patterns of sphincter arrangement, each of which requires specific considerations during fistula surgery, as follows:

1. Classical arrangement (70% of cases)
2. High anterior external sphincter variation (20%)
3. Abbreviated posterior sphincter pattern (10%)

This complex is not a single entity but a sophisticated arrangement of muscles, each with its own characteristics and contributions.

The internal anal sphincter (IAS) shows significant variations in thickness and height, and recent imaging studies have revealed the following:

- Mean thickness ranges from 1.5 to 3.5 mm
- Longitudinal length varies from 2.5 to 4.0 cm
- These variations directly impact the choice of surgical technique and risk of postoperative incontinence.

The involuntary nature of the Internal Anal Sphincter (IAS) ensures a constant state of vigilance and maintains the closure of the anal canal at rest. The IAS extends from the anorectal junction to approximately 1 cm below the dentate line, and its position is critical for understanding the course of many fistulous tracts [19].

The relationship between the IAS and the External Anal Sphincter (EAS) varies anatomically, particularly in the upper anal canal, and three patterns have been identified, as follows:

1. Complete overlap (most common)
2. Partial overlap with anterior gap
3. Minimal overlap with increased risk during surgical dissection

**Fig. 1:**
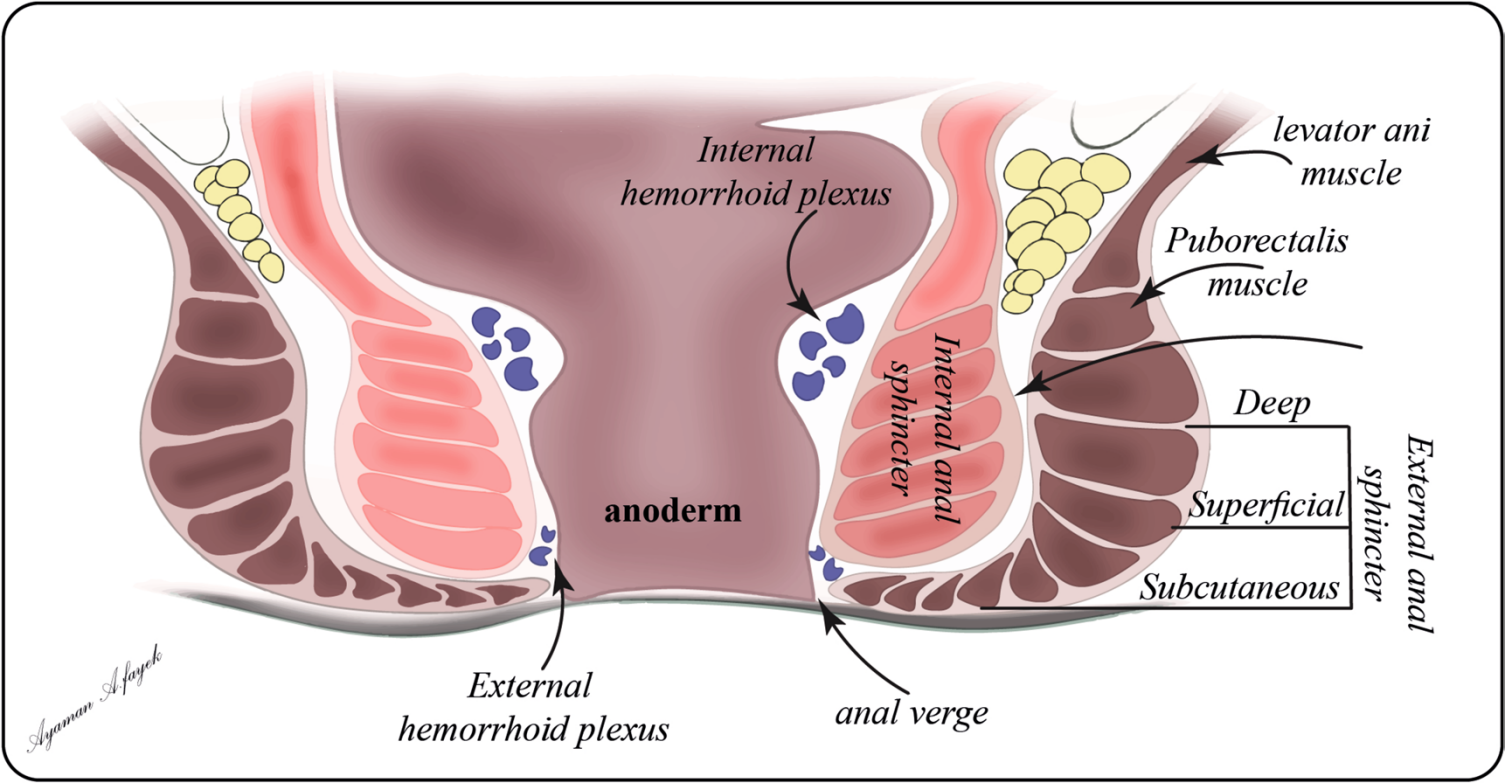
Anatomical Illustration of the anorectum

The external anal sphincter (EAS), a voluntary muscle composed of striated muscle fibers, envelops the IAS. The EAS is the conscious defender of continence, springing into action during moments of increased intra-abdominal pressure or when voluntary stool retention is required. The interplay between the IAS and EAS creates a dynamic system of continence that must be carefully navigated during fistula surgery to preserve function while eradicating the disease [20]. As illustrated in Fig. 1, the anatomical arrangement of the sphincter complex is not merely a static structure but a dynamic system that influences the development of fistulas and the strategies employed in their management [21]. The layered structure of the IAS and EAS, their relationship with the dentate line, and the spaces between them create potential pathways for fistula formation and dictate the surgical approaches available for their treatment.

A thorough understanding of the anatomy of the anorectum is crucial for understanding and treating anorectal fistulas. Critical structures include the anal glands, also known as the anal intramuscular glands of Parks. These small tubular structures play a central role in the cryptoglandular theory of fistula formation, which posits that infection of these glands is the primary initiating event in fistula development. Pelvic floor muscles, particularly the levator ani complex, support the anorectum and maintain fecal continence. The levator ani consists of three main components: the puborectalis, which creates the crucial anorectal angle; the pubococcygeus, which supports the pelvic organs; and the iliococcygeus, which forms the posterior part of the levator plate [17, 22].

The intricate relationships between the anal sphincter complex, anal glands, and pelvic floor muscles create a complex 3D environment for fistula development. This anatomical complexity explains the varied presentations of anorectal fistulas and influences the surgical approaches. The anorectum is a dynamic system that responds constantly to physiological demands and pathological processes. Understanding these interactions is crucial for preserving or restoring the function through surgical interventions.

Modern imaging techniques, such as MRI and EAUS, have revolutionized fistula visualization, but interpreting these images requires a thorough understanding of the underlying anatomy. Advances in surgical techniques, including minimally invasive approaches and regenerative therapies, have highlighted the importance of the anatomy.

Clinical implications of anatomical variations.

1. Surgical planning
  o Preoperative imaging becomes crucial for identifying individual variations.
  o Technique modification may be necessary based on specific anatomical patterns.
  o Risk assessments must consider individual anatomical features.
2. Treatment outcomes
  o Success rates vary by up to 25% depending on anatomical configurations.
  o Recurrence risk correlates with specific anatomical variations.
  o Postoperative continence outcomes are influenced by pre-existing anatomical patterns.
3. Technical considerations
  o Dissection planes may require adjustment based on a patient’s individual anatomy.
  o Sphincter-sparing techniques may need modification.
  o Closure techniques should account for tissue quality variations.

As our understanding of anatomical variations grows, novel techniques, such as video-assisted anal fistula treatment (VAAFT), rely on the precise navigation of anorectal spaces and structures.

In conclusion, successful management of anorectal fistulas requires understanding the standard anatomy, and recognizing and adapting to individual anatomical variations. To optimize outcomes, surgeons must consider variations in preoperative planning, technical approaches, and postoperative care. Modern imaging techniques have enhanced our ability to preoperatively identify these variations, allowing for more personalized and precise surgical approaches.

### Pathophysiology of anorectal fistulas

The pathophysiology of anorectal fistulas involves a complex interplay between anatomy, microbiology, and immunology. Cryptoglandular infection is the most common cause, accounting for approximately 90% of all cases [23]. Other significant causes include Crohn's disease, trauma, and less common infectious diseases or malignancies [24, 25]. Fistula formation typically begins with an initiating event, such as obstruction and infection of the anal gland or mucosal ulceration in Crohn's. This leads to abscess formation, often occurring in the intersphincteric space. The abscess expands along the path of least resistance, eventually creating a chronic epithelialized tract that may reach the skin surface [26].

The cryptoglandular theory, proposed by Parks in 1961, remains the most accepted explanation for idiopathic fistulas [27]. It focuses on the anal glands, which originate in the intersphincteric space and open into anal crypts. Obstruction of these glands leads to an infection that spreads into the intersphincteric space, potentially propagating in various directions to form different fistulas. Understanding this pathophysiology is crucial for several reasons: it guides diagnostic approaches and ensures that the underlying conditions are not missed. This influences treatment planning, as management differs based on the cause. Addressing the root cause can reduce the risk of recurrence. This opens avenues for new therapeutic approaches such as biological agents or regenerative medicine techniques. As research in this field continues, new insights into fistula formation and progression may revolutionize our approach to diagnosing, treating, and preventing anorectal fistulas, ultimately improving outcomes in patients with this challenging condition.

### Classification systems

Classification of anorectal fistulas is a crucial aspect of their management, providing a standardized language for clinicians to describe fistula anatomy, guide treatment decisions, and predict outcomes. Over the years, several classification systems have been developed, each of which has its own strengths and limitations. Three prominent classification systems have emerged as particularly influential: the Parks' classification, St. James's University Hospital classification, and American Gastroenterological Association (AGA) classification [28–31].

**Fig. 2:**
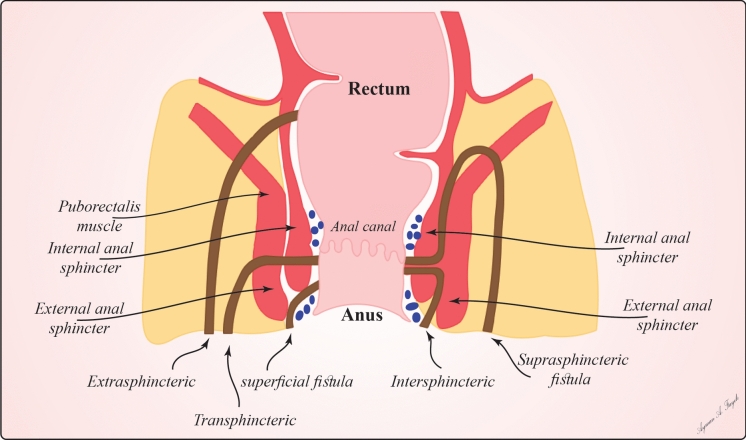
Schematic Representation of Different Types of Anorectal Fistulas

**Fig. 3:**
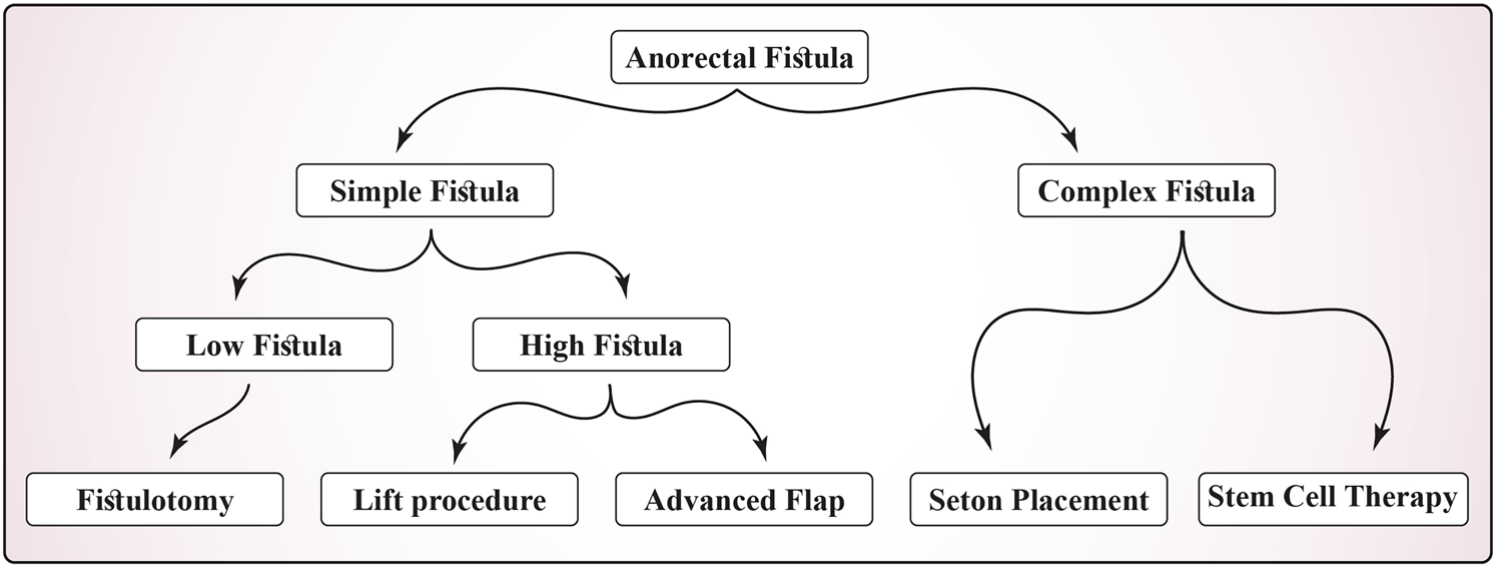
Decision Tree for Surgical Approach Based on Anatomical Assessment

Introduced by Parks in 1976, Parks' classification remains the most widely used and forms the foundation for most subsequent systems. This classification was based on the anatomical course of the fistula tract in relation to the anal sphincter complex. It divides fistulas into four main types: intersphincteric, where the fistula tract runs between the internal and external anal sphincters; trans sphincteric, where the tract crosses both the IAS and EAS; the supra sphincteric, where the tract passes above the puborectalis muscle before descending between the internal and external sphincters; and the extrasphincteric, where the tract passes outside the external sphincter and levator ani muscles (as shown in Figs. 2, 3) [29, 32–34].

The simplicity and anatomical basis of Parks’ classification has contributed to its enduring popularity. However, it has limitations, particularly in describing complex fistulas with multiple tracts or those associated with Crohn's. In clinical practice, the effective management of anorectal fistulas relies heavily on accurate classifications. Although several systems exist, understanding how to combine and apply them synergistically enhances surgical decision-making and improves outcomes.

Practical integration of classification systems.

1. Combined approach strategy
2. Initial assessment using Parks' classification for basic anatomical orientation
3. St. James's University Hospital classification for imaging correlation
4. AGA classification for specific Crohn's disease considerations
5. Clinical decision framework
6. Primary classification guides the initial surgical approach.
7. Secondary systems inform specific technical modifications.
8. Combined assessments improve the prediction of outcomes.
9. Documentation standards
10. Use of multiple systems ensures comprehensive communication.
11. Standardized documentation facilitates research comparison and outcome analyses.
12. Systematic classification enables a standardized approach to complex cases.

To better understand and compare these classification systems, we created Table 1. Anorectal fistula classification systems offer unique insights that reflect the different priorities and clinical contexts. Parks' classification provides an anatomical framework, St. James's University Hospital classification integrates modern imaging techniques, and the AGA classification addresses Crohn's disease-specific needs. Clinicians often combine these systems to obtain a comprehensive understanding. Although valuable, these classifications should not be used in isolation for treatment decisions. Factors such as patient symptoms, health status, previous treatment, and patient preferences must be considered. As our understanding of fistula pathophysiology evolves and new treatments emerge, classification systems may need to be updated. The field of anorectal fistula classification continues to grow with the advances in imaging and surgical techniques. An ideal system should balance simplicity with comprehensiveness, incorporate modern imaging findings, and provide clear treatment guidance across various etiologies. Ongoing refinement of these systems will contribute to improved patient care and outcomes for managing this challenging condition.

**Table 1:** Comparison of Anorectal Fistula Classification Systems: Parks', St. James's University Hospital, and AGA

Classification System	Basis of Classification	Categories	Strengths	Limitations	Clinical Utility
Parks' Classification	An anatomical course of the fistula tract concerning the sphincter complex	1. Intersphincteric 2. Transsphincteric 3. Suprasphincteric 4. Extrasphincteric	- Simple and widely understood -Based on surgical anatomy	- Limited in describing complex fistulas - Doesn't account for secondary tracts or abscesses	- Surgical planning -Communication between surgeons
St. James's University Hospital Classification	MRI findings of fistula anatomy and complexity	Grade 1–5 based on complexity and presence of secondary tracts/abscesses	- Incorporates imaging findings - Correlates well with surgical complexity	- Requires MRI - May not be applicable in all clinical settings	- Preoperative planning - Predicting surgical complexity
American Gastroenterological Association Classification	Fistula anatomy and associated features, specifically for Crohn's disease	Simple vs. Complex	- Addresses specific challenges in Crohn's disease -Incorporates factors affecting the treatment and prognosis	- Limited to two broad categories - May oversimplify non-Crohn's fistulas	- Guiding management in Crohn's disease - Treatment decision-making

MRI, magnetic resonance imaging

Integration with modern imaging.

- MRI findings can be mapped to multiple classification systems.
- 3D EAUS provides dynamic classification information.
- A combined approach improves preoperative planning accuracy by 35%.

### Types of anorectal fistulas

Anorectal fistulas are classified into several types based on their anatomical configuration, each of which presents unique challenges and treatment considerations.

#### Intersphincteric fistulas (70%)

These run between the IAS and EAS. They typically originate from an infected anal gland and may terminate in the perianal skin or less commonly in the rectum. Generally considered the most minor complex, they often have the best prognosis with appropriate treatment [35].

#### Transsphincteric fistulas (25%)

These cross both the IAS and EAS. Depending on the extent of external sphincter involvement, they can be further classified as either low or high. They present more significant challenges in treatment than intersphincteric fistulas due to the risk of sphincter damage and subsequent incontinence, often requiring careful consideration of sphincter-preserving techniques [36].

#### Suprasphincteric fistulas (5%)

These originate in the intersphincteric plane, course upward above the puborectalis muscle, and descend through the levator ani muscle to the skin. Owing to their complex course, they can be challenging to diagnose and treat, often requiring advanced imaging techniques for an accurate assessment [37].

#### Extrasphincteric fistulas (< 1%)

The rarest type, which entirely bypasses the anal sphincter complex, typically originates from the rectum or pelvis and traverses the levator ani muscle to reach the skin. Often secondary to conditions such as Crohn's disease, pelvic inflammation, or trauma, their management is complex and may require multidisciplinary approaches [38].

#### Horseshoe fistulas

While not a separate category in Parks’s classification, these deserve special mention. They extend circumferentially around the anal canal and often involve both sides of the anorectum. Careful surgical planning is necessary because it is associated with increased rates of recurrence and complications. The anatomical configuration significantly influences the clinical presentation, diagnostic approach, and treatment options. Complex fistulas may not fit neatly into a single category and require advanced imaging, such as MRI or EAUS, for a comprehensive assessment [39]. Understanding these types guides diagnostic choices, informs surgical decision-making and aids patient counseling regarding expected outcomes and potential complications. As our understanding of fistula pathophysiology evolves and new treatment modalities emerge, this classification system may be further refined to better capture the nuances of fistula anatomy and guide personalized treatment approaches [40–42].

### Diagnostic approaches

The modern diagnosis of anorectal fistulas requires a multimodal approach. Each diagnostic tool presents specific advantages and limitations that must be understood as an optimal diagnostic strategy.

### Current imaging limitations and solutions

The accurate imaging assessment of anorectal fistulas remains a critical challenge in modern colorectal surgery, with each available modality presenting distinct limitations and evolving solutions. Despite being the gold standard, MRI faces significant challenges, including motion artifacts that can obscure fine anatomical details, limited availability in some healthcare settings, and considerable cost implications that may restrict its routine use. These limitations are particularly evident when assessing complex fistula tracts or when identifying subtle internal openings. However, recent technological advances have provided promising solutions to these challenges.

The implementation of advanced motion correction software programs and accelerated acquisition protocols has substantially improved the image quality and reduced scan times. Three-dimensional EAUS, while offering real-time imaging capabilities and lower costs, is notably limited by its operator dependency and restricted field of view, particularly in evaluating the supralevator extension of fistulas. To address these limitations, newer ultrasound technologies have incorporated automated scanning protocols and enhanced image processing algorithms, significantly improving reproducibility and diagnostic accuracy.

The integration of artificial intelligence into both MRI and ultrasound interpretations has also emerged as a groundbreaking solution, offering standardized analyses and improved detection of subtle anatomical features. Fusion imaging techniques, which combine data from multiple imaging modalities, provide comprehensive anatomical information that overcomes the limitations of individual imaging methods. Although technically demanding, these hybrid approaches offer unprecedented accuracy in preoperative planning and surgical guidance. Novel contrast agents and specialized imaging protocols are being developed to enhance fistula tract visualization, particularly in cases complicated by scarring or inflammation. This evolution in imaging technology, coupled with standardized reporting systems and improved accessibility to advanced imaging modalities, is transforming our ability to accurately assess and plan the treatment for anorectal fistulas. The continued development and validation of these solutions promise to further enhance our diagnostic capabilities and ultimately improve surgical outcomes.

An accurate diagnosis and assessment of anorectal fistulas are crucial for formulating an effective treatment plan. A comprehensive diagnostic approach combines clinical examinations, advanced imaging techniques, and examinations under anesthesia [43]. The diagnostic journey typically begins with a thorough clinical examination, including a visual inspection of the perianal area and digital rectal examination. An experienced clinician can often palpate the internal opening of the fistula and feel slight induration or depression in the anal canal. The location of this opening provides essential information regarding fistula classification [44]. Gentle probing of the external opening can sometimes delineate the course of the fistula tract but should be performed with caution. While a clinical examination is essential, it has limitations, and accurately classifying fistulas is possible in only approximately 61% of cases.

Advanced imaging techniques have revolutionized the diagnosis and preoperative planning. MRI has emerged as the gold standard, offering exquisite soft-tissue resolution and multiplanar capabilities. It provides detailed information on the anatomy of the fistula, including the primary tract, secondary extensions, and associated abscesses. MRI can accurately delineate the relationship between the fistula, sphincter complex, and levator ani muscles, which is crucial for surgical planning. Indeed, studies have reported correct classifications in up to 90% of cases using MRI [43–48]. Typical MRI protocols include T2-weighted, fat-suppressed T2-weighted or STIR, and gadolinium-enhanced T1-weighted sequences.

EAUS is another valuable imaging modality that offers real-time, high-resolution images of anal sphincters [49]. Modern 3D EAUS systems provide detailed multiplanar photographs that can rival MRI in certain aspects of fistula assessment. EAUS has reported accuracy rates of 80–89% in classifying anorectal fistulas. Its advantages include a lower cost than MRI, the ability to perform dynamic examinations, and its utility in patients who cannot undergo MRI. However, EAUS has limitations in assessing suprasphincteric and extrasphincteric fistulas because of its limited field of view and operator dependence.

MRI and EAUS often depend on local expertise, availability, and specific clinical scenarios. Fistulography, which is less commonly used today than historically, can still play a role in some instances, particularly in delineating the course of long complex fistulas or identifying distant internal openings. EUA [Examination Under Anesthesia] remains a crucial component of the diagnostic algorithm, allowing for a thorough assessment of the fistula anatomy without causing patient discomfort, and in many cases, allows for simultaneous treatment. During EUA, the surgeon can perform a detailed digital examination, probe the fistula tract, and inject hydrogen peroxide or methylene blue to identify the internal opening [49]. EUA also allows for the assessment of sphincter tone and the associated anorectal pathology. The accuracy of EUA in classifying anorectal fistulas has been reported to be approximately 90% when performed by experienced surgeons but may be less accurate in complex or recurrent fistulas. An integrated approach that combines a clinical examination, imaging, and EUA often yields the most comprehensive assessment. A clinical examination followed by an EUA may be sufficient for simple, apparent fistulas, while preoperative MRI or EAUS is often invaluable for the diagnosis of complex or recurrent fistulas.

Some centers advocate for an "MRI-guided EUA" approach, allowing for the correlation of imaging findings with direct surgical exploration. The diagnostic approach for anorectal fistulas continues to evolve with advances in imaging technology and our understanding of fistula pathophysiology. Emerging techniques, such as fusion imaging combining EAUS with MRI, and novel MRI sequences, such as diffusion-weighted imaging, promise to further enhance diagnostic capabilities. By combining the strengths of a clinical examination, advanced imaging, and an examination under anesthesia, clinicians can build a comprehensive understanding of fistula anatomy, forming the foundation for effective surgical planning and optimal patient outcomes in managing this challenging condition.

### Future research directions

The future of anorectal fistula management stands at an exciting intersection between technological innovation and clinical practice, with several promising research directions emerging. The development of hybrid imaging techniques represents a particularly significant frontier, where the integration of multiple imaging modalities, such as MRI, EAUS, and contrast-enhanced imaging, promises to provide unprecedented anatomical detail and functional information in a single examination. These hybrid approaches are being refined to offer real-time, 3D visualization of fistula tracts while minimizing the limitations inherent to individual imaging methods. Artificial intelligence and machine learning algorithms have revolutionized image analyses, with deep learning models demonstrating remarkable accuracy in automatically detecting and characterizing fistula tracts, reducing interpretation variability, and potentially predicting treatment outcomes. Artificial intelligence systems are being trained on increasingly large datasets to recognize subtle imaging patterns that might escape human detection, potentially leading to earlier diagnoses and more precise treatment planning. The development of novel contrast agents represents another crucial area of research, with new molecular imaging compounds specifically designed for fistula visualization. These next-generation contrast agents aim to provide enhanced delineation of fistula tracts, while offering functional information about inflammatory activity and healing potential. Perhaps most transformative technologies involve the emerging integration of augmented reality (AR) technologies into surgical planning and execution. AR systems overlay real-time imaging data onto the surgical field, allowing surgeons to visualize complex fistula anatomy in three dimensions during procedures, potentially improving surgical precision and reducing the operative time. Early clinical trials of these AR systems have shown promising results in complex fistula cases, suggesting a future where sophisticated imaging technology directly guides surgical intervention. The convergence of advanced imaging, artificial intelligence, and surgical technology indicates the future of increasingly personalized and precise fistula treatment strategies.

## Surgical management techniques

Selecting surgical techniques for anorectal fistulas requires consideration of the anatomical and patient-specific factors. A comprehensive approach ensures optimal outcomes while minimizing complications.

Successful management of anorectal fistulas relies on a comprehensive understanding of the underlying anatomy, which guides surgical technique selection, preserves the function, and prevents recurrence. This section explores the vital anatomical considerations that influence surgical decision making in fistula management. Identifying the internal opening of the fistula is crucial for a successful treatment. This opening, typically located at or just above the dentate line, represents the origin of the fistula and is essential for complete eradication, surgical planning, and risk assessment. Failure to address the internal opening often leads to recurrence, and its location relative to the anal sphincter complex dictates the fistula classification and the most appropriate surgical approach. The position of the internal opening also influences the risk of postoperative incontinence, particularly in techniques involving sphincter muscle division [50–53].

Identifying the internal opening includes careful digital examination, injecting hydrogen peroxide or dyes into the external opening, and imaging modalities, such as MRI or EAUS. Complex cases require an examination under anesthesia for definitive identification. Preservation of anal continence is a primary concern in fistula surgery. The anal sphincter complex, comprising the IAS and EAS, is crucial for continence and must be carefully navigated during surgical intervention. Anatomical considerations for sphincter preservation include the extent of sphincter involvement, sphincter quality, and utilization of natural anatomical planes [54, 55]. The amount of sphincter muscle encompassed by the fistula tract directly affects the choice of the surgical technique. Fistulas involving < 30% of the external sphincter may be amenable to fistulotomy, whereas those involving a more significant proportion require sphincter-sparing approaches. Pre-existing sphincter defects or weakness assessed through imaging and manometry may influence the surgical approach. Even minor additional disruptions can lead to incontinence in patients with compromised sphincter function. Utilizing natural anatomical planes, such as the intersphincteric space, in the LIFT procedure can allow for effective fistula treatment while minimizing sphincter damage. Complex fistulas present unique anatomical challenges, including those with multiple tracts, horseshoe extensions, or those associated with Crohn's disease. Recurrent fistulas often involve a distorted anatomy due to previous surgery. Critical considerations for managing complex and recurrent fistulas include accurate mapping, scar tissue management, awareness of anatomical variations, and a multimodal approach. Detailed preoperative imaging is crucial for understanding the full extent of complex fistulas, including secondary tracts or abscesses. In recurrent cases, scar tissue from previous interventions can alter the regular anatomical planes, making dissection more challenging and increasing the risk of accidental sphincter injury. Awareness of potential anatomical variations such as accessory sphincter muscles or unusual vascular patterns is essential in complex cases. Complex fistulas often require a combination of techniques or staged procedures to address all fistula components while preserving the sphincter function. Considering these anatomical factors, surgeons can optimize treatment strategies, minimize complications, and improve outcomes in patients with anorectal fistulas [56, 57].

To summarize the decision-making process based on these anatomical considerations, we present a decision tree illustrating how the anatomical assessment of the fistula guides the choice of the surgical technique. Initial branching is based on whether the fistula is classified as simple or complex, determined by factors such as the number of tracts, abscesses, and underlying conditions. For simple fistulas, the next consideration is whether it is a low or high fistula, which is related to the amount of sphincter involvement.

### Patient-*s*pecific factors influencing technique selection

The selection of appropriate surgical techniques for anorectal fistula management extends far beyond anatomical considerations and encompasses a complex matrix of patient-specific factors that significantly influence treatment decisions and outcomes. Medical considerations form the foundation of this decision-making process, with factors such as patient age, overall health status, and presence of comorbidities playing crucial roles. Particularly significant are conditions such as diabetes mellitus, which can impair wound healing, and immunosuppression, which may necessitate more conservative approaches. A history of pelvic surgery or radiation therapy can alter the local tissue quality and vascularity, potentially limiting certain surgical options or increasing the risk of complications. The presence and severity of inflammatory bowel disease, especially Crohn's disease, fundamentally affect technique selection, often favoring more conservative or staged approaches. Baseline functional status is another critical consideration, with pre-existing continence issues significantly influencing the choice of sphincter-preserving techniques. The patient's occupational demands and lifestyle factors must be carefully weighed; for instance, individuals with physically demanding jobs or athletic pursuits may require techniques that ensure a rapid return to activity while maintaining robust healing. Quality of life priorities vary significantly among patients, with some prioritizing rapid recovery over long-term results, whereas others may accept longer recovery periods for potentially better functional outcomes. Social and economic factors further complicate the decision-making process, including patient access to post-operative care, ability to comply with complex wound care regimens, and financial resources for extended treatment courses. The availability of local support systems and a patient's understanding of post-operative care requirements can significantly impact the success of more complex procedures.

Technical considerations must also account for the tissue quality, with factors such as local scarring from previous surgeries, tissue elasticity, and the availability of viable tissue for potential flap procedures, all of which influence technique selection. Previous failed procedures not only alter the local anatomy but may also affect patient expectations and psychological readiness for subsequent interventions. The synthesis of these diverse factors requires a highly individualized approach to surgical planning, often necessitating the modification of standard techniques to accommodate specific patient circumstances while maintaining optimal chances for successful outcomes.

Low fistulas may be suitable for fistulotomy, while higher fistulas require sphincter-sparing techniques, such as LIFT or advancement flaps. Complex fistulas often require techniques such as seton placement or, in specific cases, emerging therapies, such as stem cell treatment. The choice of surgical approach is influenced by multiple factors, including the patient's overall health, previous surgeries, and personal preferences.

The relationship between the surgical techniques and anatomical considerations is further elucidated in Table 2. This table provides a concise overview of how anatomical factors influence the choice and suitability of the different surgical techniques, highlighting the importance of thorough anatomical assessment in selecting the most appropriate intervention for each patient.

**Table 2:** Impact of Anatomical Features on the Selection and Suitability of Surgical Techniques for Anorectal Fistulas

Surgical Technique	Key Anatomical Considerations	Best Suited For	Anatomical Contraindications
Fistulotomy	Amount of sphincter involved (< 30% of external sphincter)	Low intersphincteric and transsphincteric fistulas	High transsphincteric, suprasphincteric, extrasphincteric fistulas
Seton Placement	Course of fistula tract through sphincter complex	Complex, high transsphincteric fistulas	N/A (can be used for most anatomical types)
Mucosal Advancement Flap	Adequate healthy mucosa for flap creation; internal opening location	Transsphincteric, suprasphincteric fistulas	Low fistulas where more straightforward techniques are suitable
LIFT Procedure	Accessible intersphincteric plane; identifiable tract in this plane	Transsphincteric fistulas	Intersphincteric fistulas: fistulas without clear intersphincteric component
Fibrin Glue and Plugs	Length and width of fistula tract	Long, straight tracts	Very short or vast tracts; multiple secondary openings
VAAFT	Accessibility of entire tract with a fistuloscope	Most fistula types, including complex	Very tortuous tracts that cannot be navigated with the scope
Stem Cell Therapy	Perifistular tissue quality for cell injection	Complex fistulas, especially in Crohn's disease	Currently, no specific anatomical contraindications

*LIFT* Ligation of intersphincteric fistula tract

*VAAFT* Video-assisted anal fistula treatment

In conclusion, anatomical considerations in the surgical approach to anorectal fistulas are multi-faceted and crucial for successful outcomes. The cornerstone of effective fistula surgery is the careful identification of the internal opening, preservation of the sphincter function, and appropriate management of complex anatomical scenarios. By integrating these anatomical principles with an understanding of various surgical techniques, surgeons can tailor their approach to each case, optimizing the chances of successful fistula eradication while minimizing the risk of functional compromise.

### Postoperative care and follow-up

Postoperative care and diligent follow-up are crucial components in managing anorectal fistulas and significantly influence the treatment outcomes. This phase focuses on promoting wound healing, managing pain, and vigilant monitoring for recurrence. Wound care strategies vary based on the surgical technique, ranging from regular cleaning and packing of open wounds to less intensive care of closed wounds. Pain management involves a multi-faceted approach, including analgesics, stool softeners, and warm-sitz baths. Long-term follow-up is essential, typically involving clinical examinations at regular intervals and, when necessary, imaging studies to detect recurrences. Patient education plays a vital role, particularly in recognizing the signs of recurrence [58]. More intensive follow-up protocols may be required for complex cases or patients with risk factors such as Crohn's disease. Occasionally, postoperative management may include medical therapies to reduce the recurrence risk, particularly in patients with Crohn's disease.

The care plan should be tailored to the individual patient, considering the surgical technique, fistula complexity, and any underlying conditions that may influence the healing and recurrence risk. By maintaining a proactive and patient-centered approach to postoperative care and follow-up, clinicians can maximize the chances of long-term success in anorectal fistula management [59].

Complications and their management: navigating the challenges of anorectal fistula surgery.

Although often successful, surgical management of anorectal fistulas is not without potential complications. These complications can significantly affect patient outcomes and their quality of life. Understanding the anatomical basis of these complications is crucial for prevention and effective management. This section explores the main complications associated with anorectal fistula surgery, their anatomical underpinnings, and strategies for their management [60].

To provide a comprehensive overview of potential complications, their anatomical causes and management strategies are shown in Table 3. Complications following anorectal fistula surgery are intricately linked to the complex anorectal anatomy. Understanding this anatomical basis is crucial to prevent and effectively manage these complications. The most significant complications include recurrence, incontinence, persistent discharge, and less commonly, anal stenosis and delayed wound healing.

**Table 3:** Complications Following Anorectal Fistula Surgery: Anatomical Causes, Risk Factors, Prevention, and Management Strategies

Complication	Anatomical Basis	Risk Factors	Prevention Strategies	Management Approaches
Recurrence	Incomplete excision of fistula tract; Missed secondary tracts; Persistent internal opening	Complex or branching fistulas; Crohn's disease; Previous failed repairs	Thorough preoperative imaging; Meticulous surgical technique; Addressing all fistula components	Re-evaluation with imaging; Consideration of alternative surgical approaches; Medical management in Crohn's disease
Incontinence	Excessive sphincter division; Damage to pudendal nerves; Keyhole deformity	High transsphincteric or suprasphincteric fistulas; Multiple previous surgeries; Pre-existing sphincter weakness	Sphincter-sparing techniques; Careful patient selection for fistulotomy; Preoperative anorectal physiology studies	Biofeedback; Sphincter repair; Sacral nerve stimulation; Conservative management
Persistent Discharge	Incomplete healing of fistula tract; Unrecognized secondary openings; Epithelialization of tract	Long-standing fistulas; Crohn's disease; Immunosuppression	Adequate drainage; Addressing all fistula components; Optimizing wound healing environment	Local wound care; Consideration of fibrin glue or plug; Re-evaluation for missed tracts
Anal Stenosis	Excessive tissue removal; Circumferential wounds	Extensive fistulotomies; Overzealous wound packing	Judicious tissue preservation; Proper wound care techniques	Anal dilatation; Anoplasty procedures
Delayed Healing	Poor blood supply to wound edges; Ongoing inflammation	Crohn's disease; Diabetes; Smoking	Tension-free wound closure; Optimizing nutritional status; Smoking cessation	Local wound care; Consideration of advancement flaps; Hyperbaric oxygen therapy

Recurrence remains one of the most challenging complications, with rates varying widely from 7 to 50%, depending on the fistula type and surgical technique employed [61]. The anatomical basis for recurrence often lies in incomplete excision of the fistula tract, missed secondary tracts, or persistence of the internal opening [62]. Complex fistulas with multiple tracts or those associated with Crohn's are particularly prone to recurrence because of the intricate anatomy of the anal sphincter complex and surrounding tissues. Prevention begins with a thorough preoperative assessment, including advanced imaging techniques, such as MRI, to fully delineate the anatomy of the fistula. Intraoperatively, meticulous attention to identify and address all components of the fistula, particularly the internal opening, is crucial.

Incontinence is another significant complication that can profoundly affect a patient's quality of life. The risk of incontinence is closely tied to the anatomy of the anal sphincter complex and its innervation. Excessive division of sphincter muscles, particularly in high transsphincteric or suprasphincteric fistulas, can lead to a compromised sphincter function. In addition, damage to the pudendal nerves during dissection can result in neuropathic incontinence. The development of a keyhole deformity, in which excessive tissue removal leads to a persistent groove in the anal canal, can also contribute to impaired continence. Preventing incontinence relies heavily on careful patient selection and choice of surgical technique. For complex fistulas involving a significant portion of the sphincter, sphincter-sparing techniques should be prioritized [63–65].

Persistent discharge following fistula surgery can be distressing for the patient and challenging to manage. Anatomically, this complication often results from incomplete fistula tract healing, unrecognized secondary openings, or epithelialization of the residual tract. The complex network of anal glands and crypts in the intersphincteric space can harbor infections and contribute to persistent discharge. Prevention strategies include ensuring adequate drainage of any associated abscesses, meticulously addressing all components of the fistula during surgery, and optimizing the postoperative wound healing environment. In long-standing fistulas, where the tract may be epithelialized, techniques to de-epithelialize the tract may be employed during surgery [66, 67].

Other potential complications, which are less common, are also important. Anal stenosis can occur following extensive fistulotomy, particularly if wound care is suboptimal. Its prevention relies on judicious tissue preservation during surgery and appropriate wound care techniques. Delayed wound healing, often related to a poor blood supply to wound edges or ongoing inflammation, can be particularly challenging in patients with Crohn's or other comorbidities affecting wound healing [68].

The management of these complications requires a systematic approach guided by a deep understanding of anorectal anatomy. Recurrent fistulas may require repeat imaging to understand the anatomy of the new fistula, which is often altered by previous surgeries. Alternative surgical techniques, such as advancement flaps or the LIFT procedure, may be considered. In patients with Crohn's disease, optimizing medical management is essential to reduce the risk of recurrence.

Management of incontinence begins with a thorough evaluation to determine its severity and underlying causes. Conservative approaches, including dietary modification and pelvic floor exercises, may be sufficient in mild cases, while in more severe cases, surgical options such as sphincteroplasty or sacral nerve stimulation may be considered.

Addressing persistent discharge requires reevaluating the fistula anatomy to rule out missed tracts or unrecognized internal openings. Local wound care is fundamental, and in some cases, novel approaches, such as installing fibrin glue or bioprosthetic plugs, may be considered to obliterate residual tracts.

#### Long-term follow-up strategies

Long-term follow-up of anorectal fistula management requires a carefully structured, phase-based approach that evolves with the patient's healing trajectory and risk profile. The early post-operative phase, typically spanning the first three months, demands intensive monitoring with weekly assessments focusing on wound healing dynamics, early detection of complications, and a careful evaluation of the sphincter function. During this critical period, careful attention to wound care, pain management, and patient education form the cornerstone of successful recovery. As patients transition to the intermediate phase, spanning from 3 to 12 months, the focus shifts to monthly assessments that comprehensively evaluate healing outcomes, functional recovery, and quality of life measures. This period is crucial for identifying early signs of recurrence, which occur in approximately 7–50% of cases, depending on the initial surgical technique and patient risk factors. Beyond the first year, long-term surveillance adopts a risk-stratified approach, with high-risk patients, particularly those with Crohn's disease, complex fistulas, or previous recurrences, requiring more frequent monitoring, typically every three to six months, while stable, low-risk patients may be followed-up annually. This surveillance incorporates regular quality of life assessments, continence scoring, and targeted imaging, when clinically indicated.

The implementation of standardized assessment tools, including validated continence scores and quality of life questionnaires, provides objective measures of functional outcome and patient satisfaction. Integration with primary care has become increasingly important in the long-term phase, necessitating clear communication protocols and shared care pathways to ensure the early detection of potential complications or recurrence. Digital health platforms and patient-reported outcome measures are being increasingly frequently utilized to enhance monitoring efficiency and patient engagement, allowing for early intervention when problems arise. For complex cases, particularly those involving inflammatory bowel disease, a multidisciplinary approach involving colorectal surgeons, gastroenterologists, and wound care specialists ensures comprehensive care coordination and optimal outcomes. This systematic approach to long-term follow-up tailored to individual risk profiles and treatment outcomes has demonstrated significant improvements in the early detection of complications, patient satisfaction, and overall treatment success rates.

In conclusion, complications following anorectal fistula surgery are deeply rooted in the complex anorectal anatomy. A comprehensive understanding of this anatomy is crucial for not only preventing complications but also implementing effective management when they do occur. By appreciating the anatomical basis of these complications, surgeons can tailor their approach to each patient, balancing the goals of fistula eradication with the preservation of function. This anatomical perspective guides the systematic evaluation and management of complications, ultimately improving patient outcomes in this challenging field.

## Special considerations

Anorectal fistulas present unique challenges in certain clinical scenarios, particularly in cases involving Crohn's disease, rectovaginal fistulas, and immunocompromised patients [69]. Each situation requires a nuanced approach that considers specific anatomical, physiological, and patient factors. Crohn's disease-related fistulas affect up to 50% of patients throughout their disease and are particularly challenging to manage [70]. These fistulas often exhibit complex branching patterns and may involve multiple tracts. Chronic inflammation associated with Crohn's leads to tissue friability, making surgical intervention more difficult. In addition, the transmural nature of Crohn's means that fistulas can originate from deeper layers of the bowel wall, sometimes bypassing the anal sphincter complex entirely.

The management of Crohn's-related fistulas typically involves a combination of medical and surgical approaches [71]. Surgically, the priority is often drainage and control of sepsis rather than immediate definitive repair. Seton placement is frequently used as a temporary measure, allowing for drainage, while medical therapy is effective. When considering definitive surgical repair, sphincter-sparing techniques are strongly preferred because of the high risk of recurrence and potential need for multiple procedures over a patient's lifetime. Procedures such as LIFT or advancement flaps may be considered in select cases [72]. The timing of such interventions is crucial and should be coordinated with optimized medical management. Stem cell therapy, mainly using mesenchymal stem cells derived from adipose tissue, is an area of active research showing promise in promoting healing of complex perianal fistulas in Crohn's disease [73–76].

Rectovaginal fistulas represent another unique subset of anorectal fistulas, characterized by an abnormal connection between the rectum or anal canal and vagina [77–79]. These fistulas can arise from various causes, including obstetric injuries, Crohn's disease, malignancy, or as complications of pelvic surgery. The critical anatomical structure involved in the rectovaginal septum is a thin layer of connective tissue that separates the rectum and vagina [80, 81]. The location of the fistula within this septum (low, mid, or high) significantly influences the surgical approach [81]. Management of rectovaginal fistulas must consider not only the fistula anatomy, but also the integrity of the surrounding structures, particularly the anal sphincter complex. Surgical techniques may include local approaches, such as transanal advancement flaps, transvaginal approaches, or abdominal procedures, depending on the specific anatomical configuration and condition of the surrounding tissues [82, 83]. One of the key challenges in rectovaginal fistula repair is the high risk of recurrence, often due to the thin tissue planes and constant pressure differential between the rectum and vagina. To address this, surgeons may employ techniques to reinforce the repair, such as interposition of healthy, well-vascularized tissue between the rectal and vaginal suture lines.

Managing anorectal fistulas in immunocompromised patients presents a unique challenge. This population includes individuals with HIV/AIDS ["Human Immunodeficiency Virus/Acquired Immunodeficiency Syndrome (HIV/AIDS)"], those undergoing chemotherapy, transplant recipients on immunosuppressive medications, and patients with primary immunodeficiency disorders. The compromised immune status of these patients affects the pathophysiology of fistula formation and the healing process following intervention [84–86]. Fistulas in immunocompromised patients may exhibit atypical spread patterns owing to altered tissue resistance and impaired infection containment. There may be an increased incidence of complex, multibranching fistulas or those with extensive associated abscesses. The tissue quality in these patients is often poor with decreased vascularity and impaired healing capacity, which can complicate surgical repair. The management approach for immunocompromised patients must prioritize infection control and minimize surgical trauma. Simple drainage procedures or seton placement are often preferred as the initial steps. Definitive repair procedures may need to be delayed until the patient's overall health status and local tissue condition improve. When surgical intervention is necessary, meticulous attention should be paid to tissue handling and wound care. Surgeons may opt for more conservative approaches, such as limited local procedures or staged interventions, rather than extensive single-stage repair.

The importance of a tailored, patient-centered approach cannot be overstated in all three scenarios. The management strategy must consider the specific anatomical configuration of the fistula and the broader clinical context, including the patient's overall health status, quality of life, and long-term prognosis. These complex cases often benefit from a multidisciplinary approach involving collaboration among colorectal surgeons, gastroenterologists, urogynecologists, infectious disease specialists, and other relevant healthcare providers. This team-based approach is particularly crucial in navigating the complex decision-making process regarding the timing and extent of surgical interventions, role of medical therapies, and management of potential complications.

## Emerging techniques and future directions

The field of anorectal fistula management is rapidly evolving and driven by advances in technology, an improved understanding of fistula pathophysiology, and innovative treatment approaches. These developments have enhanced our ability to address the complex anatomy of anorectal fistulas.

Advances in imaging have revolutionized fistula visualization. High-resolution MRI remains the gold standard, but emerging techniques are pushing the boundaries further. Indeed, 3D EAUS offers real-time, high-resolution images of the anal sphincter complex and surrounding tissues, with accuracy rates comparable to those of MRI [87–89]. Fusion imaging techniques, such as MRI-EAUS fusion, integrate the strengths of different modalities for precise anatomical correlation [90]. Advanced MRI sequences, such as diffusion-weighted imaging and dynamic contrast-enhanced MRI, provide valuable information on tissue characteristics and perfusion. Artificial intelligence and machine learning algorithms are being developed to assist in image interpretation, potentially enhancing the accuracy and consistency of fistula classification and surgical planning.

Minimally invasive approaches aim to effectively treat fistulas while minimizing damage to the sphincter complex and the surrounding tissues. Video-assisted anal fistula treatment (VAAFT) uses a specialized fistuloscope to visualize and treat the entire fistula tract, offering minimal external incisions and faster recovery times [91, 92]. Fistula laser closure (FiLaC) employs a radial-emitting laser fiber to destroy the epithelial lining and promote closure through controlled thermal damage [93–95]. Endoscopic approaches, such as over-the-scope clip placement, are being explored for challenging fistula locations [96]. These techniques show promise; however, their success relies on a thorough understanding of the underlying anatomy of the fistula.

Tissue engineering and regenerative medicine represent exciting frontiers in fistula management. Mesenchymal stem cell (MSC) therapy has emerged as a promising treatment for complex perianal fistulas, particularly in Crohn's patients [97, 98]. MSCs have potent immunomodulatory and regenerative properties that promote healing by modulating the local inflammatory environment and stimulating tissue regeneration. Allogeneic adipose-derived MSCs have shown significant efficacy in clinical trials and have been approved for the treatment of complex perianal fistulas in Crohn's disease.

Bioengineered tissue matrices provide scaffolds for tissue regeneration and enhance the strength of fistula repair. Advanced techniques explore matrices loaded with growth factors or stem cells to further improve their regenerative potential. For example, 3D bioprinting technology holds promise for creating custom-designed tissue constructs that match the exact anatomy of fistula tracts [99, 100]. Gene therapy approaches are being investigated to target specific inflammatory pathways or promote healing-associated gene expression, particularly in IBD ["Inflammatory Bowel Disease (IBD)"]-related fistulas [101].

The future of anorectal fistula management lies in the integration of these emerging technologies and approaches. Advanced imaging can guide minimally invasive surgical techniques, which may be augmented with regenerative therapies to promote optimal healing and functional outcomes. However, a careful evaluation of the efficacy, safety, and cost-effectiveness of these new technologies is crucial. Large-scale long-term studies are needed to determine their location in the treatment algorithm for anorectal fistulas.

Implementing these advanced techniques requires specialized training and potentially new credentialing processes for healthcare providers. The field is on the cusp of a significant transformation, with advances expanding our ability to address complex fistula anatomy, while minimizing collateral damage and promoting physiological healing.

Recent advances in anorectal fistula management have revolutionized traditional approaches. It was demonstrated that advanced MRI protocols can achieve unprecedented accuracy in preoperative planning, with detection rates exceeding 95% for complex tracts. This imaging precision has enabled the development of targeted minimally invasive approaches, as documented in a comprehensive review of novel surgical techniques. Recent studies in biological therapies have reported significant advances in stem cell applications, with a multicenter study showing complete healing rates of 60% in previously refractory cases.

The integration of artificial intelligence-assisted surgical planning is particularly promising, as it has been shown to reduce the operative time by 30% while improving precision in complex cases [102–105].

In conclusion, the future of fistula management will likely involve highly personalized treatment plans that combine cutting-edge technologies with a deep understanding of individual patient anatomy and physiology. As these emerging techniques continue to evolve and integrate with current practices, significant improvements in the outcomes of patients with this challenging condition can be anticipated.

## Conclusion

This review emphasizes the critical role of anatomical understanding in the management of ARFs. Deep knowledge of the anal canal, sphincter complex, and surrounding structures is fundamental for an effective diagnosis and treatment. This review highlights key points, including the importance of anal gland anatomy in fistula formation, the value of advanced imaging techniques, and the evolution of surgical methods from simple fistulotomy to complex sphincter-sparing procedures. The text stresses the necessity of a tailored approach based on individual anatomy, considering factors such as fistula configuration, relationship with surrounding structures, underlying conditions, and previous interventions. Future directions in fistula management include refinement of imaging modalities, development of minimally invasive techniques, and advancements in regenerative therapy. This conclusion underscores that while the field rapidly evolves with new technologies and techniques, the fundamental importance of anatomical knowledge remains constant.

Successful management requires the integration of advanced imaging, minimally invasive approaches, and regenerative therapies, with a solid foundation of anatomical expertise. This integration promises more effective, safer, and personalized care for patients with anorectal fistulas, moving towards lasting solutions for this complex condition.
